# Characterizations of Pectin from *Choerospondias axillaris* Fruit Pulp: Comparison of Different Extraction Methods

**DOI:** 10.3390/foods13233920

**Published:** 2024-12-04

**Authors:** Zian Luyang, Zhibin Bu, Jijun Wu, Yuanshan Yu, Lina Cheng, Jian Peng, Yujuan Xu

**Affiliations:** 1 Sericultural & Argi-Food Research Institute, Guangdong Academy of Agricultural Sciences/Key Laboratory of Functional Foods, Ministry of Agriculture and Rural Affairs/Guangdong Key Laboratory of Agricultural Products Processing, No.133 Yiheng Street, Dongguanzhuang Road, Tianhe District, Guangzhou 510610, China; 18201320731@163.com (Z.L.); bzb81@126.com (Z.B.);; 2 School of Food Science and Engineering, Jiangxi Agricultural University, Nanchang 330045, China

**Keywords:** *Choerospondias axillaris* fruit pulp pectin, different extraction methods, physicochemical characteristics, structure, functional properties

## Abstract

Generally, the extraction method has a great influence on the quality of pectin. However, there is little study on the effect of extraction method on the properties of *Choerospondias axillaris* fruit pulp pectin (CAPP). Accordingly, the physicochemical, structural, and functional properties of CAPP extracted by hot water (HWE), hydrochloric acid (HAE), ultrasound (UAE), and ultrahigh pressure (UPE) were investigated. Among these four CAPPs, UPE had the highest yield (15.79%) and GalA content (60.44%). UAE showed the most abundant side chains and RG-I region (55.12%). The lowest molecular weight (233.13 kDa) and yield (8.64%) were found in HAE. Though HWE exhibited better yield than HAE, its Mw was the highest. Different from physicochemical characteristics, the extraction method had a small effect on the structure of CAPP. The crystalline structure and functional group composition of different CAPPs were similar, while the surface structure of UAE and UPE had irregular circular holes in comparison with HWE and HAE. Furthermore, the extraction method also showed a great impact on the function. Compared with HWE and HAE, UAE and UPE presented better thermal stability and emulsifying properties. Meanwhile, HAE and UAE showed better antioxidant ability and prebiotic properties among these four CAPPs. The above results indicated that UAE showed better yield and functional properties. Hence, ultrasound extraction could be used as an effective method to extract CAPP.

## 1. Introduction

*Choerospondias axillaris* is a deciduous tree with edible fruit [1], which is widespread in China, Nepal, Bangladesh, and Japan. Its fruit is succulent and has a sour flavor. *C. axillaris* fruit has high nutritional value, being rich in biologically active substances, such as vitamins, polysaccharides, and amino acids [2]. However, *C. axillaris* fruit is easy to deteriorate after harvesting and difficult to store for a long time. Therefore, it is often processed into fruit cake, fruit wine, and fruit juice. Moreover, *C. axillaris* fruit is also used in traditional Chinese medicine. Its dried fruit is believed to have the ability to treat palpitations, chest tightness, irritability, and insomnia [3].

Pectin is a polysaccharide, which is abundantly present in the primary cell wall and mesocosm of plants. Pectin can be used in many areas and has been widely used as an emulsifier and gelling agent. Generally, pectin has three regions, including homogalacturonan acid (HG), rhamnogalacturonan glycan I (RG-I), and rhamnogalacturonan glycan II (RG-II) [4]. The main chain of pectin is a long linear chain of galacturonic acid (GalA) covalently linked by α-1, 4 glycosidic bonds, of which HG consists of more than 100 GalA units. RG-I consists of GalA and α-1, 2-L-rhamnose, whereas RG-II consists of the main chain of GalA and four structurally different polymer side chains [5,6,7]. It is well known that pectin with a high percentage of HG regions often exhibits better viscosity and gelling properties, while pectin with a higher percentage of RG I and/or RG II regions presents the opposite characteristics [7]. Pectins from different sources usually have different structures, resulting in different characteristics [8,9,10]. Consequently, there are many studies on the structure and properties of pectin from different sources. Nevertheless, there are fewer studies on *C. axillaris* pectin. Some researchers investigated the effect of different extraction methods on the physicochemical, structural, and functional properties of *C. axillaris* peel pectin [11,12,13], while no study has been conducted on the characteristics of *C. axillaris* pulp pectin.

Most pectin is chemically extracted at high temperatures in extremely acidic environments, which have the disadvantage of inefficiency or generating large quantities of wastewater during the production process [14]. As a result, researchers have developed a number of novel pectin extraction techniques, including microwave extraction, ultrasonic extraction, ultrahigh pressure extraction and enzyme-assisted extraction. The use of ultrahigh pressure and ultrasound in the pectin extraction process allows for more efficient and lower temperature pectin extraction [15,16]. Moreover, pectin obtained by ultrahigh pressure and ultrasonic extraction usually exhibits favorable functional characteristics and biological activity [14,17]. However, the effects of ultrahigh pressure extraction and ultrasonic extraction of pectin from the *C. axillaris* fruit pulp have not been reported in terms of physicochemical, structural, and functional properties. Therefore, in this study, hydrochloric acid, hot water, ultrasound, and ultrahigh pressure were used to extract pectin from *C. axillaris* fruit pulp, and the influences of these four extraction methods on yield, molecular weight (M_W_), monosaccharide composition, structure, heat stability, emulsifying properties, antioxidant activity, and prebiotic activity of *C. axillaris* fruit pulp pectin were investigated. 

## 2. Materials and Methods

### 2.1. Materials

Fresh *C. axillaris* fruits were purchased from a local fruit factory (Guangdong Jiabao Group Co., Ltd., Chaozhou, Guangdong, China). In addition, *C. axillaris* fruit was from Raoping, Chaozhou City, Guangdong Province, and they were harvested at the company’s local plantation in Chaozhou. Galactose, glucose, fucose, arabinose, rhamnose, mannose, xylose, glucuronic acid, and galacturonic acid were purchased from Sigma-Aldrich (Shanghai, China). Trifluoroacetic acid (TFA) and Dimethyl sulfoxide (DMSO) were bought from Anpel Co., Ltd. (Shanghai, China).

### 2.2. Pectin Pretreatment

The extraction of *C. axillaris* fruit pulp pectin (CAPP) was performed according to the method by Li et al. [12]. One kilogram of *C. axillaris* fruit pulp was dried using a heat pump dryer (101-3-ABS, Shanghai Ke Heng Industrial Co., Shanghai, China) at a temperature of 40 °C until it reached a constant weight. The dried pulp was milled using a grinder and sieved into fine powder by passing it through a mesh sieve of 40. The powder was washed by adding the ethanol of 95%. The sediment was then washed with acetone, and the suspension was centrifuged to obtain the precipitation. The precipitation was dried and stored in a desiccator until it was subjected to different treatments. 

#### 2.2.1. Hot Water Extraction

The dried precipitation was mixed with deionized water, and the mixture was placed in a water bath at 90 °C for 2 h with continuous stirring (DF-101S, Shanghai Li-Chen Bangxi Instrument Technology Co., Shanghai, China). The mixture was centrifuged at 5000× *g*. Following centrifugation, the supernatant was precipitated by the addition of three volumes of 95% ethanol and placed 4 °C overnight. After dialysis, the pectin was dialyzed in distilled water and freeze-dried with a freeze dryer (ST85B3-1, Millrock Technology Corp., New York, NY, USA).

#### 2.2.2. Hydrochloric Acid Extraction

The dried precipitation was dissolved in 200 mL hydrochloric acid (pH 1.80), and the suspension was stirred and heated at 90 °C for 1 h. Subsequent extraction was performed in accordance with Section 2.2.1.

#### 2.2.3. Ultrahigh Pressure Extraction

The pectin extracted by ultrahigh pressure was carried out with a 57-L ultrahigh pressure apparatus (SHPP-57DZM-600, Sanshuihe Technology Co., Ltd., Taiyuan, China). The dried precipitation was dissolved in deionized water (1:40, *w*/*v*). The mixture was placed in polyethylene vacuum bags and treated with ultrahigh pressure of 250 MPa for 15 min. Subsequent extraction was performed in accordance with Section 2.2.1.

#### 2.2.4. Ultrasonic Extraction 

The dried precipitation was dissolved in deionized water (1:40, *w*/*v*), then the mixture was treated under ultrasonic power of 240 W by an ultrasound bath (XZY-30-900DS, Zhengzhou Shengyuan Instrument Co., Zhengzhou, China) for 30 min. The ultrasonic frequency and temperature were set as 40 kHz and 40 °C, respectively. Subsequent extraction was carried out in accordance with Section 2.2.1.

The pectins extracted with hot water, hydrochloric acid, ultrahigh pressure, and ultrasound were recorded as HWE, HAE, UPE, and UAE, respectively.

### 2.3. Physicochemical Properties of Pectin

#### 2.3.1. Determination of Pectin Yield 

The yield of extracted pectin was determined using Equation (1):

(1) $$\mathrm{Yield}\;(\%)=\frac{m_{0}}{m}\times 100\%$$

where *m*_0_ and *m* represent the weight of dried pectin (g) and AIR (g), respectively.

#### 2.3.2. Determination of Galacturonic Acid Content

The galacturonic acid (GalA) content was measured using the 3, 5-dimethylphenol method described by Gharibzahedi [18]. The absorbance for each sample was read at 520 nm with a UV-1900i (Shimadzu Corporation, Kyoto, Japan).

#### 2.3.3. Determination of Esterification Degree

The degree of esterification (DE) of CAPP was determined by the titration method by Pinheiro [19]. The pectin solution was titrated with 0.1 mol/L NaOH in the presence of phenolphthalein until the solution turned pink and remained for 30 s. The initial volume of NaOH solution consumed by the pectin was recorded as *V*_1_. Then, the solution was treated with 0.1 M NaOH and agitated for a duration of 30 min. Subsequently, the same volume of 0.1 M hydrochloric acid was introduced to eliminate the pink color of the solution. Three drops of phenolphthalein indicator were added. The solution was then titrated with 0.1 M NaOH until a stable pink color emerged and persisted for at least 30 s. The volume of NaOH solution used in this final titration was recorded as *V*_2_. The degree of esterification of the pectin was calculated using Equation (2):

(2) $$\mathrm{DE}\;(\%)=\frac{V_{2}}{\left(V_{1}+V_{2}\right)}\times 100\%$$

#### 2.3.4. Determination of Monosaccharide Composition

The monosaccharide composition of pectin samples was determined using a previous method [16]. The monosaccharide composition was determined by a Dionex ICS 5000+ system high-performance anion-exchange chromatography system (Thermo Fisher Scientific Inc., Waltham, MA, USA) according to the absolute quantitative method. The HPAEC system was equipped with a carboPacTM PA-20 anion-exchange column (3 × 150 mm, 10 μm, Dionex Corporation, Sunnyvale, CA, USA) and a pulsed amperometric detector (PAD). The chromatography employed three mobile phases: H_2_O, 0.1 M NaOH, and a mixture of 0.1 M NaOH and 0.2 M NaAc. The mass of monosaccharide was measured by standards, and the molar ratio was calculated according to the molar mass of the monosaccharide [20]. 

#### 2.3.5. Determination of Mw

Mw of CAPP was determined using the method reported by Zhong et al. [14]. For the measurement of molecular weight (Mw), a liquid phase U3000 system (Thermo, USA) was utilized with an Optilab T-rEX oscillometric detector (Wyatt Technology, Goleta, CA, USA) and a DAWN HELEOS II laser light scattering detector (Wyatt Technology, CA, USA). The concentration and information of the molecules were detected by the refractive index detector and multi-angle laser light scatterometer. This system was equipped with two size exclusion chromatographic columns (Ohpak SB-805 HQ, SB-803 HQ, 300 mm × 8 mm, Tokyo, Japan). The corresponding Mws of each component were calculated according to the Mark–Houwink equation. 

### 2.4. Structural Properties

#### 2.4.1. Fourier Transform Infrared (FTIR) Spectra

The FTIR method applied by Gharibzahedi [18]. Pectin was analyzed by FTIR spectrometer (VERTEX 70, Nicolet, Madison, WI, USA). The spectral analysis was conducted within a frequency range of 4000 to 400 cm^−1^, employing a scan rate of 32 times at a resolution setting of 4 cm^−1^. The Omnic 9.2 software was used to display the spectra.

#### 2.4.2. X-Ray Diffraction

CAPP was analyzed using an X-ray diffractometer, specifically a D8-Advance diffractometer (Bruker, Karlsruhe, Germany). The samples were scanned over a diffraction angle (2θ) range of 4°–60° using Cu-Kα radiation at 40 kV and 30 mA. 

#### 2.4.3. SEM

The pectin samples were promptly affixed to the stage using conductive double-sided adhesive tape and subsequently coated with a thin layer of gold under vacuum conditions. The gold spraying was carried out at a current of 10 mA for 1 min under vacuum conditions, followed by operation at a scanning electron microscope (S-3400N-ll, Hitachi Co., Tokyo, Japan) with an accelerating voltage of 10 kV. The surface morphology and shape of the pectin samples were obtained at high magnification.

### 2.5. Thermogravimetric (TG) and Derivative Thermogravimetric (DTG)

The thermogravimetric and derivative thermogravimetric analyses were performed using a thermogravimetric analyzer (Netzsch TG 209 F1 Libra, Selb, Germany). For the analysis, a 5 mg sample was placed onto a platinum pan. The sample was then heated in a helium atmosphere, with a flow rate of 60 mL/min, starting from 30 °C and reaching 600 °C. The heating process was carried out at a constant rate of 10 °C/min.

### 2.6. Properties of Emulsion

#### 2.6.1. Preparation of Pectin Emulsion

The emulsion was prepared according to the method described by Deng [21]. The pectin was dissolved in distilled water to prepare a 1.5% (*w*/*v*) pectin solution. The pectin solution was mixed with 2 mL soybean oil and 0.1% of antibacterial preservative (Pro Clean 300, Beyotime Biotechnology, Haimen, China). Then the mixture was sheared at 24,000 rpm for 3 min to obtain a pectin emulsion.

#### 2.6.2. Measurement of Emulsifying Activity (EA) and Emulsion Stability (ES)

The EA and ES of the pectin emulsion were analyzed based on the method by Jiao [22]. At 0 and 10 min, 10 mL of emulsion was taken from the bottom of the emulsion and mixed with 1 mL of SDS solution (0.1% wt), then the absorbance of the mixture was recorded at 500 nm using a UV-Vis spectrophotometer. The EA was calculated according to Formulas (3) and (4). The ES was computed using Formula (5):

(3) $$EA\;(\frac{m^{2}}{g})=\frac{2T}{(1-\varphi )C\times 0.1}$$

(4) $$T\;(m^{-1})=\frac{2.303A_{0}D}{L}$$

(5) $$ES\;(\min)=\frac{{A\;}_{0}}{A_{0}-A_{10}}$$

where *T*, *φ*, *C*, 0.1, *D*, and *L* represent turbidity (m^−1^), fraction of oil phase forming emulsion (0.2), 10,000 g/m^3^, conversion factor, dilution factor (100), and optical range (0.01 m), respectively. *A*_0_ and *A*_10_ were the absorbance of the diluted emulsion at 0 and 10 min, respectively.

#### 2.6.3. Droplet Size

To measure the droplet size of the pectin emulsion, the emulsion was dispersed in water, and the test was initiated with a total coloration between 8 and 12%. A laser particle size analyzer (ZEN3700, Malvern, UK) was used to detect the surface area average diameter (d_3,2_) of the pectin emulsion.

#### 2.6.4. Zeta Potential

The zeta potential of the pectin emulsion droplet was measured based on the pendant drop method using a nanoparticle sizer (ZEN3700, Malvern, UK). The pectin emulsion was diluted 100-fold with deionized water.

### 2.7. Biological Activities 

#### 2.7.1. Antioxidant Activities

##### ABTS Radical Scavenging Activity

A 7 mM ABTS solution was prepared by dissolving ABTS in distilled water, followed by the addition of potassium persulphate to a final concentration of 140 mM. The pectin solution was allowed to react with the ABTS mixture in the dark at room temperature for 20 min. Following the reaction, the absorbance of the mixture was measured at 734 nm. The ABTS radical scavenging activity of the pectin sample was computed using Equation (6):

(6) $$\mathrm{ABTS}\;\mathrm{radical}\;\mathrm{scavenging}\;\mathrm{activity}\;(\%)=\frac{\left(A_{1}-A_{0})-(A_{i}-A_{j}\right)}{\left(A_{1}-A_{0}\right)}\times 100\%$$

where *A*_1_, *A*_0_, *A*_i_, and *A*_j_ represent the absorbance of the control group, reagent blank group, sample group, and sample blank group, respectively.

##### Ferric-Reducing Antioxidant Power (FRAP) Assay

To assess the ferric-reduction capacity of the pectin sample, 0.3% potassium ferricyanide was added to the pectin solution and incubated at 50 °C for 20 min. Following incubation, the reaction mixture was treated with 10% trichloroacetic acid and 0.3% ferric chloride solution. The absorbance of the mixture was measured at 700 nm. The ferric-reduction ability of pectin was quantified in terms of milligrams of Trolox equivalent per gram of the pectin sample.

The prebiotic activity of pectin was assessed according to the method by Peng [23]. *Lactiplantibacillus plantarum*, *Lactobacillus rhamnosus*, and *Limosilactobacillus fermentum* were used in this study. These three probiotics were incubated for 12 h in MRS medium at 37 °C for activation. After activation, probiotics were centrifuged and added to the non-carbohydrate base medium (0.2 g/L peptone, 0.2 g/L yeast extract, 2 g/L triammonium citrate, 5 g/L CH_3_COONa, 0.2 g/L MgSO_4_·7H_2_O, 0.05 g/L MnSO_4_·4H_2_O, 2 g/L K_2_HPO4, 1.08 g/L Tween80, pH = 5.9) at 2% (*v*/*v*) of the amount. Glucose and inulin were added to the carbohydrate-free basal medium as positive controls, respectively. The prebiotic activity of pectin was evaluated by measuring the populations of probiotics at 0 h, 12 h, and 24 h post-incubation. The populations of probiotics were enumerated using the plate counting method, and the probiotic count was expressed as colony-forming units per milliliter (CFU/mL). 

### 2.8. Statistical Analysis

All tests were performed in triplicate, and results were presented as the means ± SD using SPSS Statistic 27.0 software. The obtained data were subjected to assess the differences between samples with *p* < 0.05 considered significant. 

## 3. Results and Discussion

### 3.1. Physicochemical Characteristics

**Figure 1:**
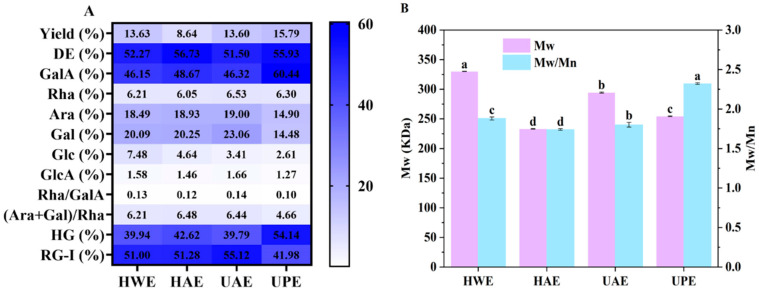
Extraction yield (**A**), DE (**A**), monosaccharide composition (**A**), and molecular features (**B**) of CAPP. In each figure, different letters represent significant differences (*p* < 0.05).

The extraction methods of pectin often impact its physicochemical characteristics [24]. Consequently, the effect of different extraction methods on the yield of CAPP was initially assessed. The yield differences mainly depended on the different extraction methods of CAPP. As shown in Figure 1A, the yield of CAPP ranged from 8.64% to 15.79%. The CAPP extracted by hydrochloric acid (HAE) exhibited the lowest yield. According to previous studies, pectin could degrade in acidic environments, and smaller fraction precipitates could not be fully obtained during pectin extraction, resulting in the lowest yield [25]. However, the CAPP extracted by ultrahigh pressure (UPE) showed the highest yield, 45.28% higher than that of HAE. Meanwhile, the yield of CAPP extracted by ultrasound (UAE) was also higher than that of HAE. Ultrahigh pressure and ultrasound could promote plant cell wall destruction and accelerate the dissolution of pectin substances [18], resulting in UPE and UAE exhibiting higher yields than HAE. 

All the CAPPs showed a relatively high DE in the range of 51% to 57% (Figure 1A), thus these CAPPs were identified as high methoxy pectin (>50%). Compared with HWE and UAE, UPE and HAE showed higher DE. Ultrasound-induced β-elimination reactions led to the breakage of the pectin side chain, resulting in lower DE values for UAE [26]. In contrast, ultrahigh pressure exposed the pectin to more methyl groups, leading to UPE having a higher DE value [14].

In addition, the extraction methods also had great influence on the Mw of CAPP. As shown in Figure 1B, CAPP extracted by hot water (HWE) presented the highest Mw (330.09 kDa), which was 12%, 30%, and 42% higher than that of UAE, UPE, and HAE, respectively. The results indicated that ultrasound, ultrahigh pressure, and hydrochloric acid extraction could reduce the Mw of CAPP to varying degrees, suggesting that both mechanical action and acid degraded the Mw of CAPP [27]. The reaction conditions of UAE and UPE were milder than those of HAE, particularly with lower temperatures and shorter processing times, which resulted in a reduction in depolymerization of CAPP [28]. Zhang et al. [7] stated that Mn is the number average molecular weight, which is the statistical average of the molecular weights of all the polymer chains in a sample and is determined by measuring the number of molecules. The Mn value represents the smaller molecular portion of the polymer for the same mass-weight. The weight average molecular weight (Mw) accounts for the size of individual chain molecular weights, reflecting the larger molecular portion of the polymer when determining the average molecular weight. The Mw/Mn ratio was used to measure the width of the molecular weight distribution of pectin; the index closer to 1 (Figure 1B) indicates a narrower distribution of pectin and better homogeneity [29]. The Mw/Mn of all CAPPs was higher than 1.0, indicating that CAPP had a wide molar mass distribution [25]. Among these CAPPs, UPE showed the highest Mw/Mn ratio (2.32), suggesting that it had the largest dispersibility. 

Furthermore, the monosaccharide composition of different CAPPs was analyzed (Figure 1A). All pectins contained higher levels of GalA and the same types of neutral sugars, including rhamnose (Rha), galactose (Gal), glucose (Glc), and arabinose (Ara). Ara (14.90–19.00%) and Gal (14.48–23.06%) were the main neutral monosaccharides for all CAPPs. The difference in Glc was mainly attributed to the fact that Glc originated from residual soluble sugars, which are non-pectin polysaccharides that remain following the extraction process [30]. However, the extraction method had a significant influence on the monosaccharide content of CAPP. UPE had the highest GalA (60.44%) content coupled with the lowest Gal (14.48%) and Ara (14.90%) concentrations, indicating that the neutral sugar side chain regions of UPE were shorter than those of other CAPPs. This might be due to the cleavage of the neutral sugar chain caused by ultrahigh pressure. 

Additionally, among these four CAPPs, HAE, UAE, and HWE presented lower HG coupled with higher RG-I content (Figure 1A), suggesting that they had a longer branching structure and shorter main chains. Ultrasound, hydrochloric acid, and hot water extraction were able to cause degradation of the GalA backbone of pectin and increase the ratio of RG-I regions [31]. The ratio of Rha/GalA indicated changes in the main chains of pectin, and the (Gal + Ara)/Rha ratio reflected the changes in pectin neutral sugar side chains and the average size of the neutral sugar side chains within the RG-I area [32,33]. There were no significant differences in the main chains of these four CAPPs, while the neutral sugar side chains of UPE showed a wide difference compared to the other CAPPs. Meanwhile, the lowest (Gal + Ara)/Rha ratio was found in UPE, suggesting that ultrahigh pressure could break the side chains of CAPP, leading to pectin with shorter side chains and fewer branched chains [33]. 

### 3.2. Structural Characteristics

#### 3.2.1. XRD

**Figure 2:**
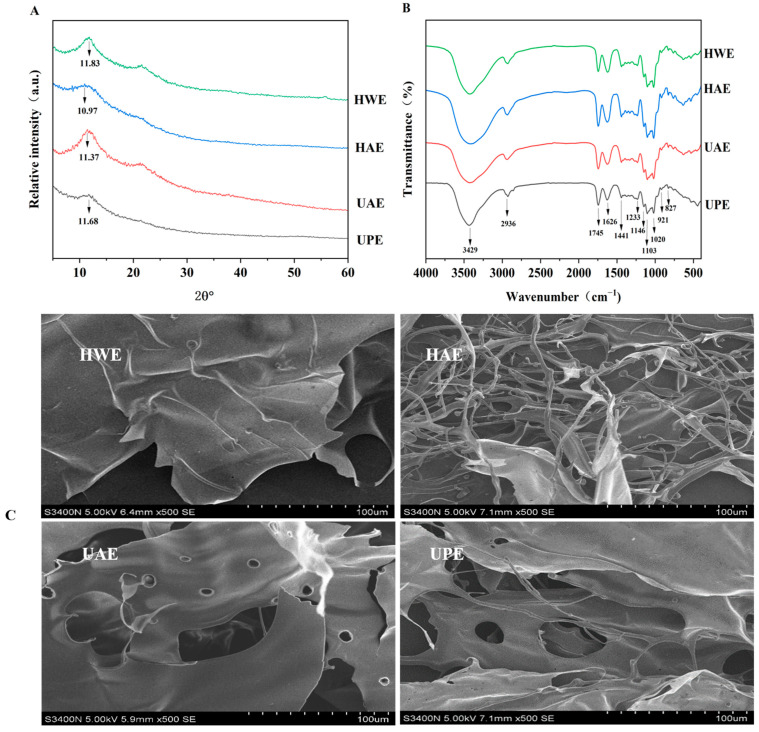
XRD spectra (**A**) and FTIR spectra (**B**), and SEM images (**C**) of CAPP.

In addition to studying the effects of extraction methods on the physicochemical properties of CAPP, the influences of extraction methods on the structural characteristics were also investigated. According to Figure 2A, the XRD spectra showed two broad diffraction peaks for all the CAPPs, indicating that the CAPP had an amorphous or semi-crystalline crystal structure. This structure is due to hydrogen bonding interactions between the hydroxyl groups of the pectin chains. Compared with HWE and UAE, the diffraction peaks of UPE and HAE became flatter at 11.6°. Ultrahigh pressure and acid disrupted the crystal structure, leading to peak changes in the XRD patterns of UPE and HAE. The formation of unformed crystalline structures might be due to the fact that ultrahigh pressure resulted in the failure of the side chains to entangle [34].

#### 3.2.2. FTIR

The functional group composition of different CAPPs was similar to a great extent (Figure 2B). The absorption peak around 3429 cm^−1^ was found in all the CAPPs, which was attributed to the O-H stretching vibration caused by hydrogen bonding [34]. The peak at 2930 cm^−1^ was related to C–H stretching vibrations of CH_2_ and CH_3_ [19]. Peaks at 1745 cm^−1^ and 1626 cm^−1^ resulted from C=O stretching vibrations of the esterified (–COOR) and unesterified (–COO-) groups, respectively [11]. The vibration of the esterified (–COOR) group at 1745 cm^−1^ was more pronounced in the case of high esterification pectin. Consequently, the absorption peaks at 1745 cm^−1^ of HAE, UPE, and UAE were higher than 1626 cm^−1^, indicating that they had a high DE. The absorption peak at 1441 cm^−1^ was related to the bending vibration of C-H, while the absorption band within the range of 1300–1000 cm^−1^ could be ascribed to the fingerprint spectrum of pectin [10,35]. Bands at 1103 cm^−1^ and 1020 cm^−1^ were associated with rhamnogalacturonan glycans, stretching of O–H bending, C–O–C glycoside rings, and C–O bonds in COOH. The bands between 1200 cm^−1^ and 1000 cm^−1^ were within the range for identification of GalA and pyranose in CAPPs [17,36]. The presence of a peak at 921 cm^−1^ indicated the presence of a D-glucopyranosyl group. The peak at 827 cm^−1^ was caused by the α-1,4 glycosidic bond, as the backbone of RG-I type is composed of l-rhamnose and d-galacturonic acid through the α-1,4 glycosidic bond [37].

#### 3.2.3. Microstructure

Figure 2C shows the SEM images of HAE, HWE, UAE, and UPE at a magnification of 500×. HWE showed a smooth surface structure and continuous flake morphology, which corresponded to the fluffy surface characteristics of the pectin. HAE presented a slender, disorganized filament morphology, indicating that hydrochloric acid led to the destruction of the structure of CAPP. Jiao et al. [22] used hydrochloric acid extraction to isolate natural polysaccharides from pumpkin, and the SEM images showed that the pectin had both flake and rod-like structures, which is similar to the shape in our study. UAE had a wrinkled surface with a lamellar structure, and some small and irregular circular holes were present on its surface, which were caused by the destructive mechanical effect of cavitation on the cell walls of the plant matrix [32]. The appearance of macropores in the lamellar structure of pectin by UAE was consistent with the findings of Wang et al. [11]. The lamellar structure of UPE contained larger pores than that of UAE, which might be attributed to the fact that ultrahigh pressure had a stronger mechanical effect on pectin compared with ultrasound. 

### 3.3. Thermal Stability

**Figure 3:**
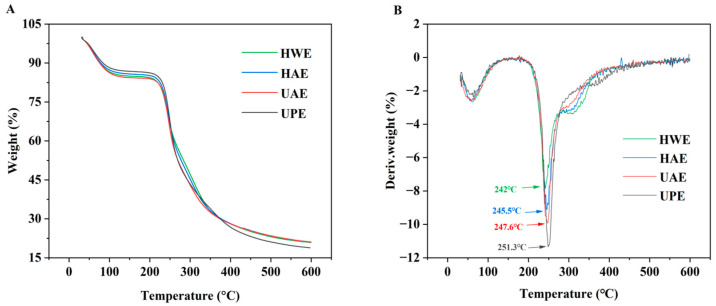
TGA curves (**A**) and DTG curves (**B**) of CAPP.

As shown in Figure 3A, the TG curves of different CAPPs were all divided into three phases: the first stage (20–170 °C), the second stage (200–380 °C), and the third stage (380–600 °C). In the first stage, all the CAPPs exhibited a weight loss of about 13–15%, indicating that the evaporation of absorbed or free water was lost in the pectin. Subsequently, the mass loss of pectin was very significant in the second stage, with a loss of about 63–68%. The mass loss of pectin was due to the pyrolytic decomposition of pectin chains and cleavage of hydrogen bonds [17]. Finally, the mass loss of pectin in the third stage was very small, with the final pectin mass remaining in the range of 18–21%. This result was attributed to the loss of the pectin carbon-containing skeleton and the degradation of the polymer backbone [38], producing a mixture of carbon and hydrocarbons as residue. 

The DTG curve represents the thermal degradation velocity of the detected samples and is the first derivative of the TG curve [39]. The first inflection point occurring around 50 °C was related to water loss. The second inflection point, close to 245 °C, was caused by the degradation process of the GalA backbone in pectin [40]. The degradation of polysaccharides proceeded through decarboxylation and deacidification processes, with various gaseous products changing to ultimately solid charcoal as the end product [37]. The maximum weight loss of UPE was observed at 251.3 °C (Figure 3B), and it might be related to its high GalA content [41]. Hence, it could be concluded that UPE had the best thermal stability.

### 3.4. Emulsifying Properties 

**Figure 4:**
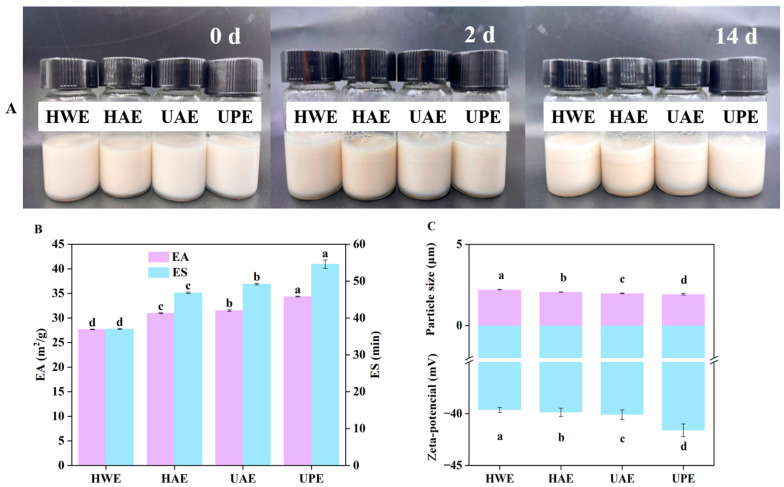
The storage stability (**A**), EA (**B**), ES (**B**), particle size (d_3,2_) (**C**), and zeta potential (**C**) of CAPP. In each figure, different letters represent significant differences (*p* < 0.05).

As can be seen from Figure 4A, HWE and HAE emulsions showed phase separation at 2 d. After 14 d of storage, all CAPP emulsions exhibited varying degrees of phase separation and the UPE emulsion showed the least degree of separation, followed by the UAE emulsion. Meanwhile, the EA and ES of HAE, UAE, and UPE emulsions were both higher than those of the HWE emulsion (Figure 4B). Pectin enhanced the emulsification properties of the emulsion by reducing the interfacial tension between the water and oil phases, and pectin with lower Mw was more effective [33]. Compared with HWE, HAE, UAE, and UPE had lower Mw. The reduction of Mw facilitated the partial exposure and adsorption of hydrophobic proteins to the oil–water interface, maintaining the stability of the emulsion; hence, their emulsions had higher values of ES [42]. Furthermore, UPE had higher DE, which enhanced intra- and intermolecular hydrophobic interactions and cross-linking of the emulsion to form thicker layers, resulting in a higher ES in the UPE emulsion [43]. Accordingly, the UPE emulsion exhibited the highest EA and ES. This result was due to ultrahigh pressure having a positive effect on the unfolding of these protein macromolecules, leading to the exposure of a large number of hydrophobic groups. Furthermore, ultrahigh pressure increased the uptake of protein molecules at the oil/water interface and reduced the size of emulsion droplets through protein structural unfolding [44]. Moreover, the emulsion droplet size is one of the key factors influencing the ES of emulsions. Emulsions with good ES usually have a small particle size to reduce gravitational emulsification [45]. Figure 4C shows that the particle size of CAPP emulsion was in the micron range, and the particle sizes of HAE, UPE, and UAE emulsions were significantly smaller than that of the HWE emulsion, suggesting that HAE, UPE, and UAE emulsions had better ES in comparison with the HWE emulsion. In addition, zeta potential is also an important factor affecting the ES [17]. The small particle size of the emulsion resulted in higher absolute values of zeta potential, which generated strong electrostatic repulsion during the dispersion process to enhance the ES through the creation of repulsive interactions between the particles [46]. The zeta potentials of UPE (−41.60 mV), UAE (−40.10 mV), and HAE (−39.87 mV) emulsions were all lower than that of HWE (−39.63 mV) emulsions, which is consistent with their ES. Ultrahigh pressure induced various physical, chemical, or biological changes in food products by affecting hydrogen bonding, covalent bonding, and van der Waals forces between molecules [33]. These changes led to the exposure of negatively charged groups on the pectin surface, which reduced the zeta potential of the pectin emulsion. The above results indicated that the UPE emulsion exhibited better emulsifying properties than other CAPP emulsions.

### 3.5. Antioxidant Capacity

**Figure 5:**
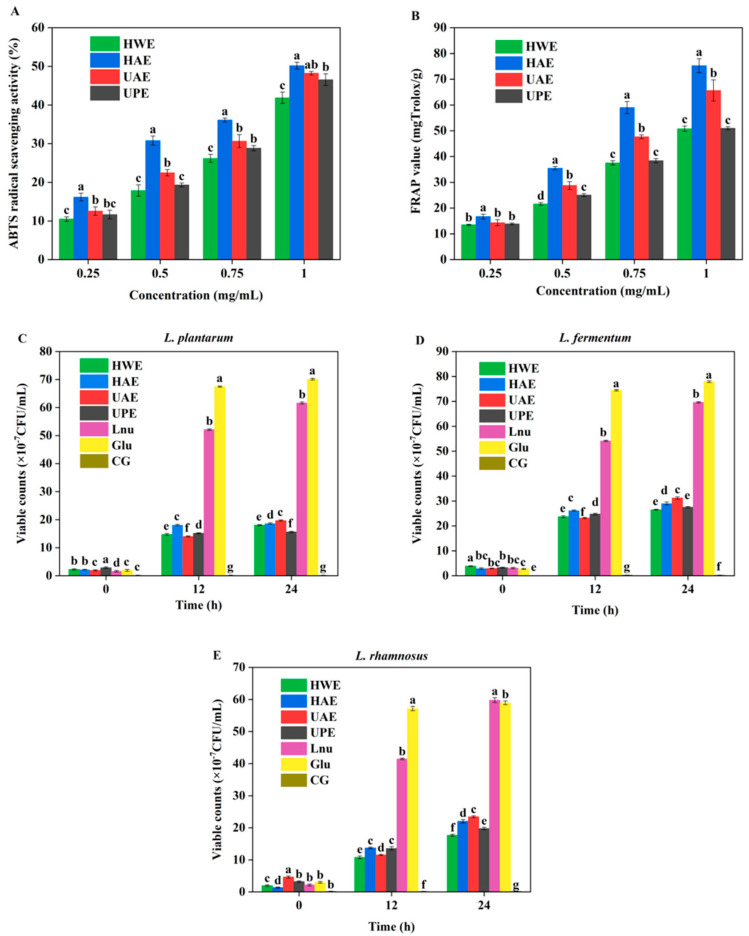
The antioxidant capacity and prebiotic activity of CAPP. ABTS radical scavenging activity (**A**), ferric reducing antioxidant capacity (**B**), viable counts of *L. plantarum* (**C**), viable counts of *L. fermentum* (**D**), and viable counts of *L. rhamnosus* (**E**). In each figure, different letters represent significant differences (*p* < 0.05).

The antioxidant capacity of CAPP was analyzed by the ABTS and FRAP methods in Figure 5A,B. The scavenging capacity of all the CAPPs for these radicals was concentration-dependent in the range of 0.25 mg/mL to 1 mg/mL. Among these four CAPPs, HAE showed the highest radical scavenging capacity, followed by UAE, UPE, and HWE. As the concentration of the CAPP solution was 1 mg/mL, the ABTS radical scavenging capacity of HAE was 50.21%. Based on previous studies, the radical scavenging capacity of pectin had a close correlation with its Mw. HAE had the lowest Mw, which might be a reason for its highest radical scavenging capacity [41]. In addition, FRAP is also a common method to evaluate the antioxidant capacity of a sample. Since the reaction environment of this method is acidic, the effect of some endogenous interfering factors can be reduced. The CAPP showed significant reducing power in a concentration-dependent manner. Similar to the results of ABTS radical scavenging capacity, the reducing ability of HAE was always higher than that of other CAPPs in the concentration range from 0.25 to 1 mg/mL. Therefore, it could be concluded that HAE had the best antioxidant capacity of all the CAPPs. UAE exhibited better reducing ability compared to UPE and HWE, which might be attributed to the potential of ultrasound to stimulate pectin production of hydrogen or electron donors [47]. The ABTS antioxidant capacity of pectin from pulp was higher than that of peel at the same concentration. However, the antioxidant mechanism of CAPP was complex, and further studies were required to investigate the interactions between CAPP and other components during the antioxidant process.

### 3.6. Prebiotic Activity

Pectin is a type of polysaccharide that can improve the proliferation of probiotics [41]. As can be seen in Figure 5, the population of the four probiotics on carbohydrate-free base medium exhibited no change, indicating that this medium could be used to evaluate the prebiotic properties of CAPP. In basal medium supplemented with carbohydrate, probiotics grew rapidly within 12 h, whereas the growth rate of probiotics started to slow down within 12–24 h. The addition of various pectins to the growth medium resulted in significantly lower levels compared to the medium supplemented with glucose after 24 h [48]. This trend was similar to our study, where CAPP was weaker than glucose in terms of prebiotic activity. Among these three probiotics, CAPP had the best capacity to promote the growth of *L. fermentum* (Figure 5D). Utilization of polysaccharides by probiotics depends on the Mw and monosaccharide composition of polysaccharides [29]. In general, pectin with a low Mw could be more easily transported and metabolized by the probiotics [24]. Therefore, UAE presented better ability to promote the growth of probiotics (Figure 5C–E). Han et al. reported that probiotics were more likely to utilize pectin with a larger RG-I region [41]. UAE exhibited a higher domain than the HAE in RG-I, resulting in UAE having a higher number of viable counts at 24 h. This demonstrated that UAE had superior prebiotic activity in comparison with HAE. Moreover, Lee et al. [49] suggested that the structure of polysaccharides influenced the growth of probiotics. The Molokhia leaf polysaccharide with a high RG-I showed better prebiotic activity, which is consistent with our study.

## 4. Conclusions

In this study, the extraction method had a great effect on the yield of CAPP, with the highest and lowest yield obtained in UPE (15.79%) and HAE (8.64%), respectively. Meanwhile, the extraction method also influenced other physicochemical properties of CAPP. Though all CAPPs belonged to high methoxyl pectin (DE > 50%), there were differences in DE among these four CAPPs. Compared with UAE and HWE, higher DEs coupled with lower Mw were observed in HAE and UPE. Moreover, UPE and UAE exhibited the highest GalA (60.44%) and RG-I contents (55.12%), respectively. Based on the above physicochemical properties, UAE presented better antioxidant capacity and prebiotic activity. Regarding the structure of CAPPs, the crystallization degree and functional groups of CAPPs were little influenced by extraction methods. However, there were differences in the morphology of the four CAPPs. In summary, the extraction methods had a greater influence on the physicochemical and functional properties of CAPP, while they had a lesser impact on the structure of CAPP. The CAPP extracted by ultrasound not only exhibited a higher yield, but also had better emulsifying properties, better antioxidant ability, and prebiotic capacity. The findings of this study provide valuable insights into CAPP extraction, and the characteristic properties of pectin can be modulated through different extraction methods.

## Data Availability

The data presented in this study are available on request from the corresponding author.
